# Acute Diffuse Peritonitis Caused by Urinary Retention: A Rare Case of Gangrenous Cystitis

**DOI:** 10.1155/2018/4948375

**Published:** 2018-06-06

**Authors:** Konstantinos Kotoulas, Chrysostomos Georgellis, Engkin Kigitzi, Apostolos Liappis, Marios Spounos, Vasiliki Tsalkidou, Emmanouil Patris

**Affiliations:** Department of Urology, University Hospital of Alexandroupolis, 681 00 Alexandroupolis, Greece

## Abstract

Gangrenous cystitis is an umbrella term encompassing conditions from necrosis of the mucosa and submucosa to necrosis of the entire bladder wall that can result in acute peritonitis. Timely diagnosis is challenging as the symptoms are nonspecific and resemble other conditions such as cystitis. We report a case of gangrenous cystitis in a 63-year-old woman who was diagnosed with peritonitis preoperatively by a CT scan of the abdomen. Overdistension of the bladder due to chronic urinary retention was the primary cause. The patient underwent partial cystectomy, excising nonviable detrusor with preservation of the trigone and ureters, but conclusively succumbed. Etiopathology, symptoms, and treatment of this rare disease are also considered.

## 1. Introduction

Gangrenous cystitis is associated with a very poor prognosis, characterized by excessive morbidity and a death rate that reaches the high percentage of 35% [1, 2]. Diabetes mellitus is a common predisposing factor mainly via overdistension of the bladder wall. Accumulation of oxidative stress products during prolonged hyperglycemia causes decompensation of the bladder tissues and function. In the advanced stage of diabetes mellitus, chronic urinary retention is frequently observed due to detrusor hypocontractility [3–5].

In this case report, we present a case of gangrenous cystitis involving the entire bladder wall. This evolved as acute abdomen and was the outcome of longstanding urine retention, initially managed only with indwelling bladder catheterization.

## 2. Case Report

A 63-year-old woman was transferred to our department from the internal medicine clinic of the hospital with a diagnosis of acute abdomen due to possible rupture of the bladder.

The patient was admitted in the internal medicine clinic three days earlier due to acute abdominal pain. She had a known medical history of uncontrolled type 2 diabetes and cirrhosis of the liver with extensive ascites. The bladder had been drained and urinary retention was observed (over 2 liters of urine). The white blood cell count was 21300, C-reactive protein (CRP) was 14,83 mg/dl, and procalcitonin was 1,1 ng/ml. Intravenous empiric antibiotic treatment with ciprofloxacin (800 mg/day) and amikacin (1000 mg/day) was immediately initiated, with good recovery until the third day.

**Figure 1:**
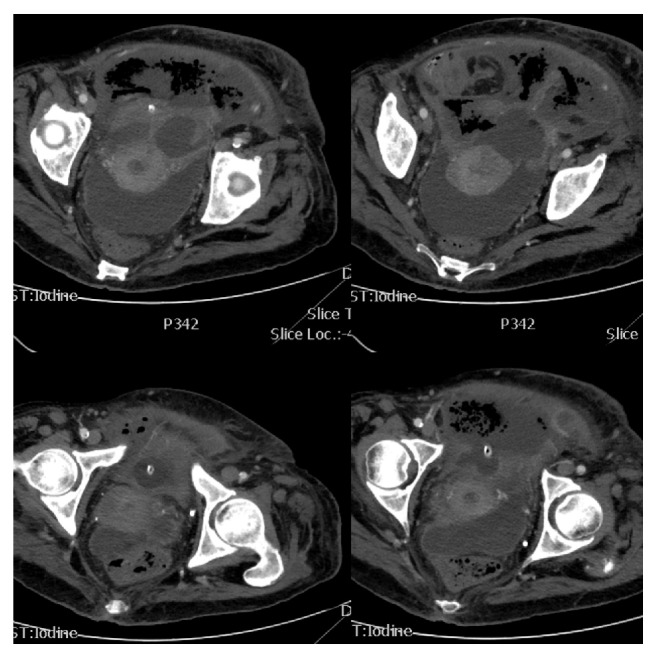
Abdominal CT scan images revealing the presence of gas within the anterior bladder wall and retropubic space. The anterior bladder wall is not enhanced by contrast material.

**Figure 2:**
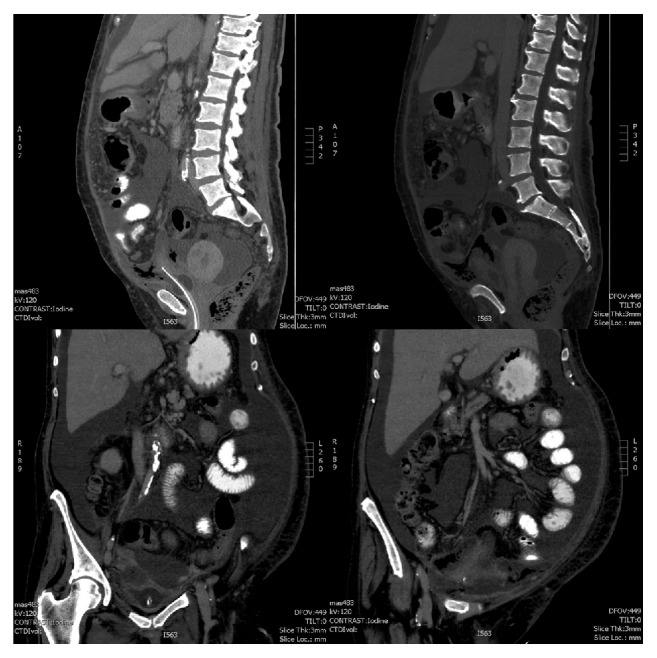
Coronal/sagittal abdominal CT scan images suggesting bladder rupture and diffuse peritonitis.

**Figure 3:**
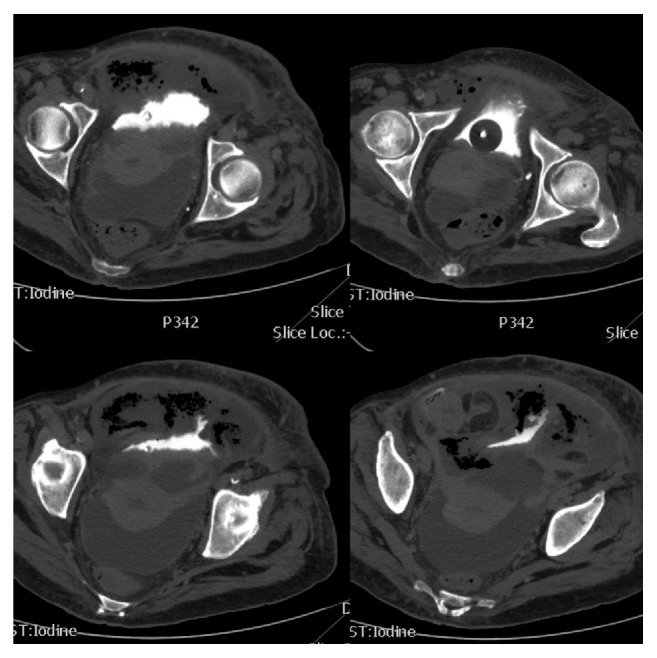
CT cystography revealed extravasation of contrast solution in the peritoneal cavity.

On day 3, the patient presented rebound tenderness, involuntary guarding, and a completely rigid “washboard” abdomen with percussion tenderness. Bowel sounds were absent. She was haemodynamically unstable. Blood pressure was 85/42 mmHg and heart rate was 114 beats per minute. Urine analysis was normal and urine culture was negative. The blood findings were as follows: WBC was 11,900 and CRP was 8,35 mg/dl. Although there was amelioration in the blood tests (as compared with baseline values), the clinical symptoms and the condition of the patient deteriorated. The computed tomography (CT) scan of the abdomen indicated presence of gas within the anterior bladder wall. The latter was not enhanced with contrast material, indicating necrosis (Figures 1 and 2). Instillation of contrast solution in the bladder through the indwelling catheter during CT revealed extravasation in the peritoneal cavity (Figure 3). Based on these results, emergency surgery was decided.

**Figure 4:**
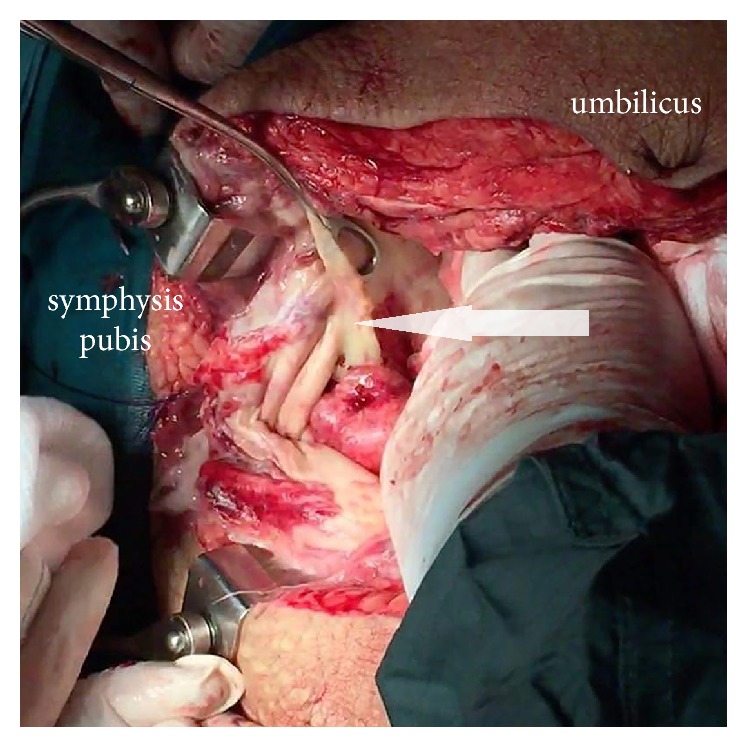
Extensive necrosis of perivesical fat (arrow) and presence of pus in the retropubic space.

**Figure 5:**
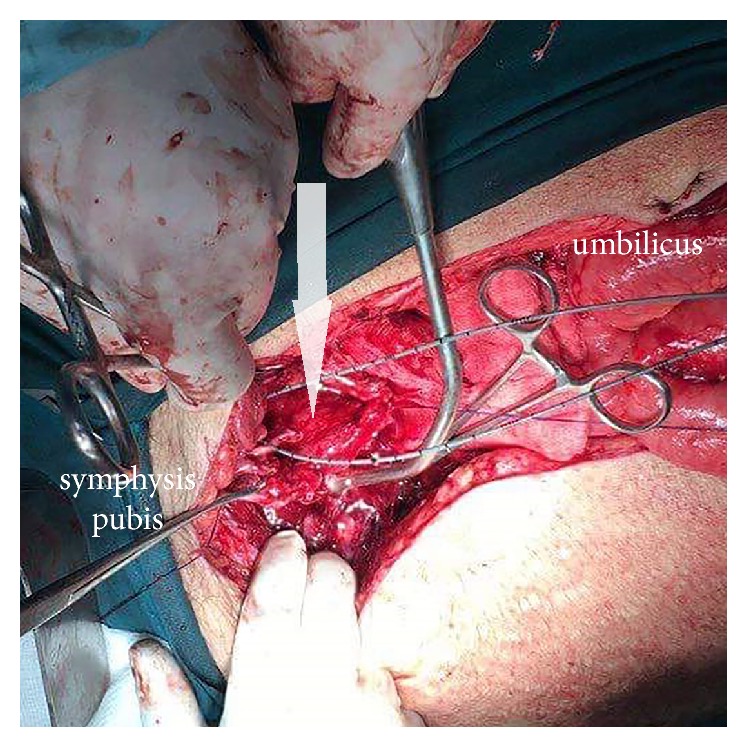
Preservation of both ureters and trigone (arrow) after debridement of the necrotic tissue.

**Figure 6:**
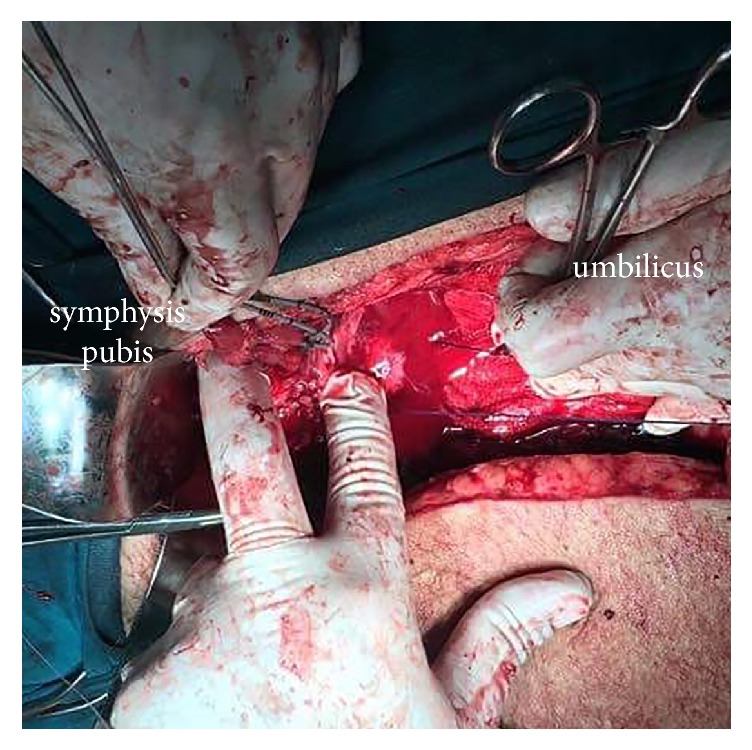
Closure of the bladder using the viable tissue from the posterior wall and trigone.

During laparotomy, we initially encountered extensive necrosis of the perivesical fat with presence of pus in the retropubic space. After the incision of the bladder, full thickness necrosis of the wall was revealed, with the exception of the anatomical area of the trigone. A partial cystectomy with debridement of the necrotic tissue and preservation of both ureters was decided (Figures 4–6). The abdominal cavity was further explored and peritoneal lavage was performed due to the presence of diffuse peritonitis. A suprapubic catheter and two surgical drains (one in the hepatorenal fossa and the other in the rectouterine pouch) were used.

The patient was transferred to the intensive care unit in a critical condition and succumbed 12 hours later due to multiple organ dysfunction and septic shock. Histology revealed extensive necrosis of the entire bladder wall.

## 3. Discussion

Numerous factors have been proposed in literature as predisposing to gangrenous cystitis, thus making etiological diagnosis challenging for the physician [6, 7].

Most commonly, primary causes are categorized as direct and indirect [8]. The first category includes agents targeting directly the vesical mucosa cells. Among others, chemicals (cyclophosphamide and tris(hydroxymethyl)aminomethane (THAM)), radiation, calculi, and widespread infection have been suggested [9].

On the other hand, blood flow pathology is thought to indirectly provoke bladder wall necrosis. This is more frequently attributed to chronic urine retention with concomitant overdistension, whereas trauma to the pelvis or pelvic vascular supply, extravesical pressure and circulatory obstruction (mainly attributed to pelvic malignancies), prolonged labor, and blockage of arterial or venous channels (by either infection or emboli) have also been described [10]. Additionally, hydrodistension (which was a common treatment strategy for interstitial cystitis) and BOO (bladder outlet obstruction) due to benign prostatic hyperplasia have been reported in the literature as causes of bladder necrosis [11, 12].

Interestingly, in the vast majority of the cases, the bladder trigone is viable. This can be possibly explained by the fact that this anatomic area is additionally irrigated by arterial branches supplying the ureters and prostate, maintaining higher blood supply compared to the rest of the bladder [1].

Most importantly, clinical presentation of gangrenous cystitis is characterized by nonspecific symptoms. Suprapubic pain, frequency and dysuria, lower abdominal discomfort, pain referred to the distal urethra, microscopic or macroscopic hematuria, pyuria, or urosepsis appertains to differential diagnosis of a variety of conditions, thus delaying timely diagnosis.

Unfortunately computed tomography, which is the most useful diagnostic tool, is usually requested only when the patient presents with signs and symptoms of acute abdomen. However, cystoscopy could also serve as a useful diagnostic tool, especially since this provides a unique opportunity of direct visualization and collection of biopsies and urine cultures [7].

With respect to the treatment, the earliest the diagnosis is suspected, the better the outcome will be for the patient. Conservative management with intravenous antibiotics, fluids, and drainage of the bladder is rarely indicated, except in cases with early disease stages and stable patients [13]. Conversely, the vast majority of the patients described in literature have been managed with laparotomic total or partial cystectomy, according to the intraoperative macroscopical status of the bladder.

## 4. Conclusion

It is undoubtedly true that bladder necrosis is uncommon but this medical condition requires vigilance by physicians, especially in the presence of predisposing factors and clinical deterioration. We report this case to highlight a rare but serious complication of long-term untreated chronic urinary retention in a poorly controlled diabetic patient.

## References

[1] Ballas K., Rafailidis S., Pavlidis T. (2007). Gangrenous cystitis. *International Urogynecology Journal*.

[2] Sataa S., Habiba M., Emna M. (2013). Urinary peritonitis caused by gangrenous cystitis. *La Tunisie Médicale*.

[3] Daneshgari F., Liu G., Birder L., Hanna-Mitchell A. T., Chacko S. (2009). Diabetic bladder dysfunction: current translational knowledge. *The Journal of Urology*.

[4] Xiao N., Wang Z., Huang Y., Daneshgari F., Liu G. (2013). Roles of polyuria and hyperglycemia in bladder dysfunction in diabetes. *The Journal of Urology*.

[5] Christ G. J., Hsieh Y., Zhao W. (2006). Effects of streptozotocin-induced diabetes on bladder and erectile (dys)function in the same rat in vivo. *BJU International*.

[6] Charra B., Hachimi A., Sodki M., Gueddari H., Benslama A., Motaouakkil S. (2008). Urinary peritonitis caused by gangrenous cystitis. *Signa Vitae*.

[7] White M. D., Das A. K., Kaufman R. P. (1998). Gangrenous cystitis in the elderly: Pathogenesis and management options. *British Journal of Urology*.

[8] Cristol D. S., Greene L. F. (1945). Gangrenous cystitis. Etiologic classification and treatment. *Surgery*.

[9] De Rosa A., Amer T., Waraich N., Bello A., Parkinson R. (2011). Gangrenous cystitis in a 42-year-old male. *BMJ Case Reports*.

[10] Rai R., Sikka P., Aggarwal N., Shankaregowda S. A. (2015). Gangrenous cystitis in a woman following vaginal delivery: an uncommon occurrence - A case report. *Journal of Clinical & Diagnostic Research*.

[11] Grossklaus D. J., Franke J. J. (2000). Vesical necrosis after hydrodistension of the urinary bladder in a patient with interstitial cystitis. *BJU International*.

[12] Stirling W. C., Hopkins G. A. (1934). Gangrene of the bladder. Review of two hundred seven cases; report of two personal cases. *The Journal of Urology*.

[13] Hinev A., Anakievski D., Krasnaliev I. (2010). Gangrenous cystitis: Report of a case and review of the literature. *Urologia Internationalis*.

